# Cognitive rehabilitation: Literature review based on levels of evidence

**DOI:** 10.1590/S1980-57642009DN30300012

**Published:** 2009

**Authors:** Patricia Regina Manzine, Sofia Cristina Iost Pavarini

**Affiliations:** 1 Student in Nursing of the Federal University of São Carlos, São Paulo, SP, Brazil. FAPESP Grantholder.; 2 Nurse, Associate Professor of the Department of Nursing and Post-graduate Program in Nursing of the Federal University of São Carlos, São Paulo, SP, Brazil. Coordinator of the Graduate Course in Gerontology.

**Keywords:** Elderly, dementia, Alzheimer’s disease, rehabilitation

## Abstract

The aim of this study was to review the scientific publications on cognitive rehabilitation in Alzheimer’s disease by year published and methodology employed. The principles of systematic review by the Brazilian Cochrane Center were used. Reviews conducted by this Center were identified together with those held on the LILACS and Medline scientific databases. Nine levels of evidence were considered for analysis and a total of 37 articles were found. The results showed a growing number of publications from 2001 onwards, with majority being published early this decade. Few studies have been published on cognitive rehabilitation, with an average of three articles published per year during the study period (1985–2008). The highest levels of evidence were observed in the more recently published studies. Cognitive rehabilitation can yield greater benefits in rehabilitating patients when associated with other forms of intervention. The latest studies demonstrating greater scientific evidence concluded that results remain limited and that further studies on the topic are needed.

## Abstract (pt)

O objetivo foi revisar publicações científicas sobre reabilitação cognitiva na doença de Alzheimer segundo ano de publicação e rigor metodológico. Foram utilizados pressupostos da revisão sistemática do Centro Cochrane do Brasil. Foram identificados estudos de revisão já realizados por este Centro e incluídos estudos das bases LILACS e Medline. Nove níveis de evidência foram considerados para análise. Foram encontrados 37 artigos. Os resultados mostram uma crescente incidência de publicação a partir de 2001, com maior concentração no início desta década. São poucas ainda as publicações sobre reabilitação cognitiva, sendo uma média inferior a três artigos por ano no período estudado (1985–2008). Os maiores níveis de evidência foram observados nas publicações mais recentes. A reabilitação cognitiva quando em associação com outras intervenções pode apresentar maiores benefícios na reabilitação dos pacientes. Os estudos mais atuais e de maior evidência cientifica apontam que os resultados ainda são limitados e novas investigações são necessárias.

## Introduction

The different dementia types all have the inherent characteristic of hampering the ability to learn new concepts and knowledge (Brandt and Rich, 1995). Alzheimer’s disease (AD) is the leading type of dementia in terms of number of cases diagnosed, and the disease has been identified as a looming public health issue.

One of the forms of Non Pharmacologic Treatment available is Neuropsychological Rehabilitation, an approach which encompasses a combination of psychotherapy, the therapeutic environment, family learning groups, schemes to instruct patients and cognitive rehabilitation. These proposals are all practiced and based on a multidisciplinary context.

Cognitive rehabilitation constitutes one of the components of neuropsychological rehabilitation (Prigatano, 1997; Ávila and Miotto, 2002). Its main objective is to empower patients and their family members to live together, and to cope with, lessen or overcome the deficiencies and cognitive changes caused by neurologic lesions (Wilson, 1996; Ávila and Miotto, 2002). The focus of the majority of interventions proposed is to stimulate memory more effectively (Caliman and Oliveira, 2005). Cognitive rehabilitation “*... involves identifying and guiding individual goals and needs, where this process calls for strategies to obtain new information or compensatory mechanisms such as the use of memory aids”* (Clare and Wood, 2008, pg.2).

When used alone, these interventions have proven to be effective in many cases by avoiding or restricting the use of drugs, yet when combined with psychotropic medication, their benefits are potentially greater and can allow medication to be reduced or withdrawn altogether (Engelhardt et al., 2005). In addition, a balanced diet, physical exercises and supervision of daily activities are conducive to cognitive, behavioral and psychological rehabilitation (Bottino et al., 2002).

Thus, caring for AD patients entails a systematic and organized method, in a bid to provide individualized care focusing on the individual and group solutions. The execution of each step in assisting to care for AD patients implies evidence-based practice both in terms of the data gathered from the demented patient as well as the clinical decisions on the most efficacious treatment intervention (Galvão CM, Sawada NO, Rossi LA., 2002). The study of evidence-based practices first emerged in 1990 in the United Kingdom, the United States and Canada. In Brazil, this approach was first adopted in the medical community within the country’s larger states and in São Paulo, Rio de Janeiro and Rio Grande do Sul State universities (Galvão C.M., Sawada N.O., Mendes I.A.C., 2003).

Evidence-based practice emerged with the aim of reaching consensus on the most relevant clinical data drawn from the results of studies and from information available on data bases, thus enabling explicit and criteria-based decision-making on specific care provided to individual patients or patient groups (Driever M.J., 2002).

The hierarchical organization of scientific evidence is dictated by the type of study design employed, i.e. of the methodological approach applied in the study (Humpris D., 1999). A classification which determines the quantitative and qualitative structure of studies is based on the categorizing of *The Cochrane Collaboration*, an international network which develops and disseminates systematic reviews on the effects of health interventions. Founded in 1993 in Oxford, this group comprises nine centers distributed world wide, one of which is the Cochrane Center of Brazil located in the city of São Paulo.

The methodology of the Cochrane Center of Brazil considers eight levels of evidence: Level 1, Systematic review and Meta-analysis of controlled studies; Level 2, Randomized clinical trials; Level 3, Cohort studies; Level 4, Case-control studies; Level 5, Case series studies; Level 6, Case studies; Level 7, Research in animals and Level 8, Opinion of respected authorities/specialists (Higgins JPT, Green S., 2008).

Implementation of evidence-based practice enables the quality of care given to patient and family to be improved since this practice has a direct bearing on clinical decisions. Moreover, the professional also needs to develop the necessary skills and know-how to obtain, interpret and integrate evidence derived from studies based on the patient’s data and clinical observations.

In a bid to find elements which can contribute to the implementation of public policies on care in demented elderly based on more explicit and qualified scientific decisions, this study sought to review scientific publications by assessing year of publication and levels of evidence on the theme of cognitive rehabilitation in elderly individuals with Alzheimer’s disease.

## Methods

An analytical bibliographic review was conducted based on the principles of the quantitative method of investigation. This study was supported by the funding body – Fundação de Amparo à Pesquisa do Estado de São Paulo (FAPESP).

The principles of the systematic review of the Cochrane Center of Brazil were used, whose objective is to pool and critically assess studies published on specific themes, determining the best levels of evidence. The first step of this study was to identify the Systematic Reviews conducted by the Cochrane Center of Brazil. For the target theme, the full Systematic Review entitled “*Cognitive Rehabilitation and Cognitive Training for early-stage Alzheimer’s disease and vascular dementia*” was found on La Biblioteca Cochrane Plus and was duly included in this study along with its online references. Subsequently, studies retrieved from the LILACS and Medline primary data bases (1966–1996) and (1997–2008) were included, respectively. These data bases were chosen for their wide scope of national and international scientific articles available in the health area, and for their powerful search tools.

To perform the search, the key search words were first defined according to the Virtual Health Library (http://www.bireme.br/php/index.php) at the DeCS link – Health Terminology (http://decs.bvs.br/) using the structured vocabulary in three languages (Portuguese, Spanish and English). The search strategy employed the terms “rehabilitation”, “cognitive therapy”, “combined therapy”, “Alzheimer’s disease”, “elderly” and “elderly aged 80 years or older”.

The Boolean Logic Operators “OR” – union or addition function, and “AND” for intersection of these terms, were added to connect the key words and synonyms of each language.

### Outline of search strategy

The inclusion criteria of articles comprised: publications between January 1985 and December 2008, theme of cognitive rehabilitation in the context of dementia (Alzheimer) in the elderly, and presentation in the form “Full text free”. Nine levels of evidence were considered for analysis. Given the current divergence in the concepts of Systematic Review and Literature Review, we elected to differentiate these two methodologies for the purposes of analysis, by assigning them separate categories. This gave the following nine levels: Systematic review and Meta-analysis, Review of the literature, Randomized clinical trial, Cohort study, Case-controlled study, Case series studies, Case-study, Research in animals, and Opinion of specialist.

## Results

Table 1 presents the 37 articles matching the study inclusion criteria. From among these (Systematic Review included), 15 were reference studies retrieved by the Systematic Review, 18 were articles found on the Medline data base (1997–2008) and three were found on the LILACS data base. No articles from the Medline data base (1966–1996) met the inclusion criteria.

**Table 1:** Articles published, by level of evidence.

Systematic review and meta-analysis			
2008	Cochrane Review	Clare L, Woods B^6^	Cognitive rehabilitation and cognitive training for early-stage Alzheimer's disease and vascular dementia
Literature review			
2001	Cochrane Review	Bird M^20^	Behavioural difficulties and cued recall of adaptive behaviour in dementia: experimental and clinical evidence
2002	LILACS	Ávila R, Miotto E^3^	Neuropsychological rehabilitaion of memory's impairments in patients with Alzheimer's disease
2002	Cochrane Review	Wilson A^21^	Towards a comprehensive model of cognitive rehabilitation
2006	Medline	Burns A, Brien JO^22^	Clinical practice with anti-dementia drugs: a consensual statement from British Association for Psychopharmacology
2006	Medline	Sitzer DI, Twamley EW, Jeste DV^23^	Cognitive training in Alzheimer's disease: a meta-analysis of the literature
2008	Medline	Hogan DB et al.^24^	Diagnosis and treatment of dementia: approach to management of mild to moderate dementia
Randomized clinical trial			
1999	Medline	Corbeil RR et al.^25^	Intervention effects on dementia caregiving interaction
2000	Cochrane Review	Quayhagen et al.^26^	Coping with dementia: evaluation of four nonpharmacologic interventions
2001	Medline	Davis et al.^27^	Cognitive intervention in Alzheimer disease: a randomized placebo-controlled study
2001	Cochrane Review	Koltai t al.^17^	Influence of anosognosia on treatment outcome among dementia patients
2003	Cochrane Review	Spector et al.^19^	Efficacy of an evidence-based cognitive stimulation therapy programme for people with dementia
2004	Medline	Chapman SB et al.^28^	Effects of cognitive-communication stimulation for Alzheimer's disease patients treated with donepezil
2004	Medline	Olazarán et al.^29^	Benefits of cognitive-motor intervention in MCI and mild to moderate Alzheimer's disease
2005	Medline	Onder G et al.^30^	Reality orientation therapy combined with cholinesterase inhibitors in Alzheimer's disease: randomized controlled trial
2005	Medline	Bening et al.^31^	Cognitive rehabilitation combined with drug treatment in Alzheimer's disease patients: a pilot study
2008	Medline	Meguro M et al.^32^	Comprehensive approach of donepezil and psychosocial interventions on cognitive function and quality of life for Alzheimer's disease: the Osaki-Tajiri Project
Cohort study			
2008	Medline	Gil P et al.^33^	Variability in the diagnosis and management of patients with Alzheimer's disease and cerebrovascular disease
Case control study			
1996	Cochrane Review	Panza et al.^34^	A rehabilitation program for mild memory impairments
2001	Cochrane Review	Zanetti et al.^35^	Effectiveness of procedural memory stimulation in mild Alzheimer's disease patients: a controlled study
2001	Cochrane Review	Moore S et al.^36^	Memory training improves cognitive ability in patients with dementia
2002	Cochrane Review	Kixmiller JS^37^	Evaluation of prospective memory training for individuals with mild Alzheimer's disease
2006	Cochrane Review	Knapp et al.^38^	Cognitive stimulation therapy for people with dementia: cost-effectiveness analysis
2007	Medline	Matsuda O^39^	Cognitive stimulation therapy for Alzheimer's disease: the effect of cognitive stimulation therapy on the progression of mild Alzheimer's disease in patients treated with donepezil
			
Case series study			
1991	Cochrane Review	Backman L. et al.^40^	The generalizability of training gains in dementia: effects of an imagery-based mnemonic on face-name retention duration
1996	Cochrane Review	Hofmann et al.^41^	Interactive computer-based cognitive training in patients with Alzheimer's disease
1997	Cochrane Review	Zanotti et al.^42^	Procedural memory stimulation in Alzheimer's disease: impact of a training programme
2002	LILACS	Bottino et al.^8^	Cognitive rehabilitation in patients with Alzheimer's disease - work's report in multidisciplinary team
2002	Medline	Clare L et al.^43^	Relearning face-name associations in early Alzheimer's disease
2002	Medline	Farina E, Fioravanti R, Chiavari L, et al.^18^	Comparing two programs of cognitive training in Alzheimer's disease: a pilot study
2003	Medline	Mahendra N, Arkin S^44^	Effects of four years of exercise, language, and social interventions on Alzheimer discourse
2007	Medline	Baldelli et al.^45^	Occupational therapy and dementia: the experience of an Alzheimer special care unit
Case study			
1987	Cochrane Review	Hill et al.^46^	Imagery mnemonic training in a patient with primary degenerative dementia
2001	Cochrane Review	Clare L et al.^16^	Long-term maintenance of treatment gains following a cognitive rehabilitation intervention in early dementia of Alzheimer type: a single case study
2003	LILACS	Avila R^47^	Neuropsychological rehabilitaion results in patients with mild Alzheimer's disease
2003	Medline	Clare L et al.^48^	Cognitive rehabilitation as a component of early intervention in Alzheimer's disease: a single case study
Opinion of specialist			
2002	Medline	Burns AS^49^	Meaningful treatment outcomes in Alzheimer's disease

The data found revealed that a mean of approximately 2.64 articles were published per year over the period spanning 1985 to 2008. Regarding the distribution of annual publications, the standard deviation was 1.94, a figure which denotes data spread centered around the mean (Pagano and Gauvreau, 2004).

The data showed that publications on cognitive rehabilitation remain low, with a mean of less than three articles published annually for the study period. In line with that proposed by other authors, there is a need for further studies on this subject seeking more in-depth evidence.

As shown in Table 2, the highest prevalence of articles was observed in 2002 (19%) followed by 2001 (n=6 or 16%), 2004 and 2008, both with four studies (11% each).

**Table 2:** Relationship between number and year of studies based on search methodology.

Year	Number of studies	%
1987	1	2.70
1991	1	2.70
1996	2	5.41
1997	1	2.70
1999	1	2.70
2000	1	2.70
2001	6	16.22
2002	7	18.92
2003	4	10.81
2004	2	5.41
2005	2	5.41
2006	3	8.11
2007	2	5.41
2008	4	10.81
	**37**	**100%**

No studies were found for the years not listed above.

A growing rate of publication on the subject was observed after the turn of the twenty first century, with articles found for each year thereafter (2000, 2001, 2002, 2003, 2004, 2005, 2006, 2007 and 2008), representing the opposite pattern to the preceding nineteenth century during which, articles presented greater time intervals between publications (1987, 1991, 1996, 1997 and 1999), thus demonstrating the growing importance and concern of scientific output on the topic of care for AD patients and those involved (caregivers, family members, researchers) over recent years. The results indicate a trend toward more frequent studies from 2001 onward.

**Figure 1:**
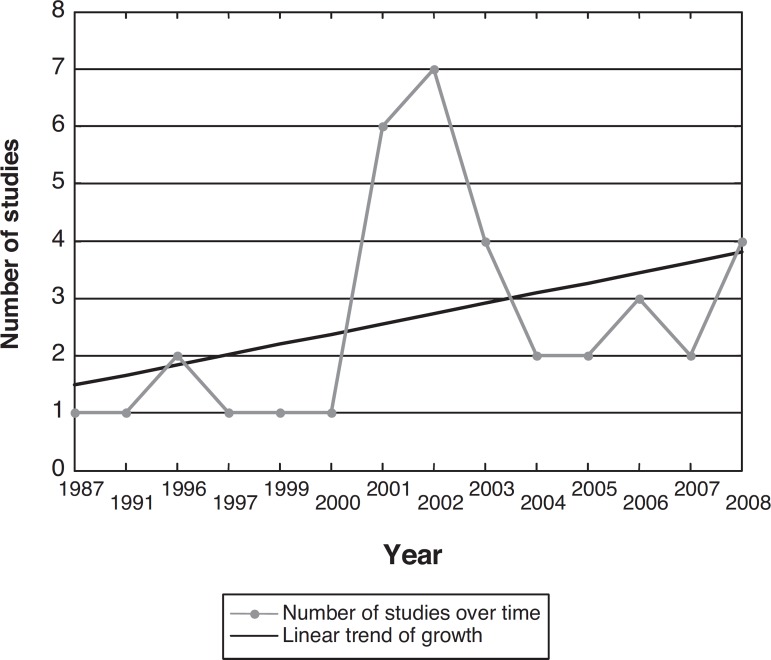
Relationship between number and year of studies found based on the search methodology.

The data show a linear growth trend in the number of studies published each year, as illustrated in Figure 1.

It is important to note that, of the six articles published in 2001, five were from the same specific period and that the present study found no reason for the high number of publications in 2002.

In terms of levels of evidence, of the 37 studies found, a mean of approximately 4.12 studies per level of evidence were obtained, with a respective standard deviation of approximately 3.58.

One study presented the highest level of epidemiological evidence from the scientific literature, namely, the Systematic Review study, where this accounted for 2.7% of the articles. The same statistical proportion of 2.7% was seen for Cohort studies and Opinion of Specialist levels.

In decreasing order of methodological merit, after the Systematic Review studies, the studies with the greatest number of quantitative findings were as follows: the Literature Review level with 16% (N=6), Randomized clinical trials with 27% (N=10), Case-Control study (N=6), accounting for 16%, and Case Series studies representing 22% of the total (N=8).

**Figure 2:**
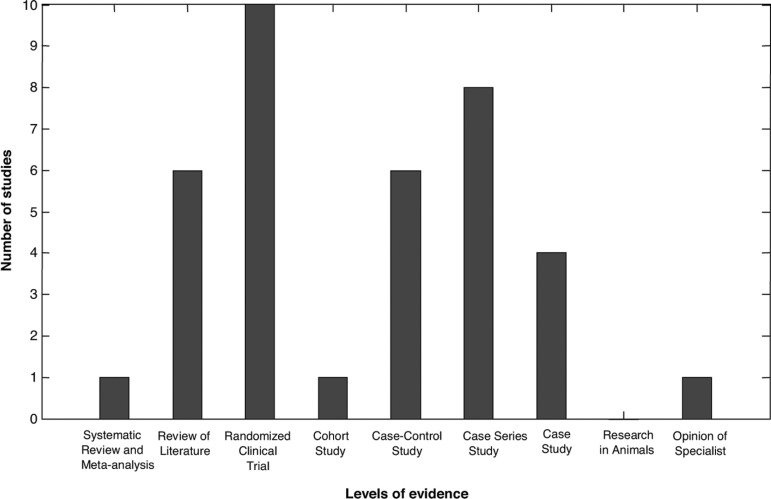
Relationship between number and level of evidence of studies found based on search methodology.

The Case study articles represented 11% of the sample found (N=4), as shown in Table 3 and Figure 2. In addition, no articles for the Research in Animals level were found (N=0).

**Table 3:** Relationship between number and levels of evidence of studies found based on search methodology.

Levels of evidence	Number of studies	%
Systematic review or meta-analysis	1	2.70%
Review of the literature	6	16.22%
Randomized clinical trial	10	27.03%
Cohort study	1	2.70%
Case-control study	6	16.22%
Case series study	8	21.62%
Case study	4	10.81%
Research in animals	0	0.00%
Opinion of specialist	1	2.70%
	**37**	**100%**

Overall, the scientific output for the subject of cognitive rehabilitation in AD patients presented a balanced disTable tribution of evidence levels of greater and lesser research relevance. However, articles employing better planned and designed scientific methodologies are needed based on the investigations currently available on the subject (Opie et al., 1999; Clare et al., 2001; Koltai et al., 2001; Farina et al., 2002; Spector et al., 2003; Clare L., Woods B., 2008).

The data obtained revealed that few studies on cognitive rehabilitation were published in the twentieth century. Studies published during this period included: one Randomized Clinical Trial (1999), one Case Control study (1996), three Case Series studies (1991, 1996 and 1997) along with the earliest study found (1987) which was a Case Study.

The results demonstrated that the only article with a high level of evidence (Systematic Review) was published in 2008. The first Review of the Literature study was produced in 2001 (N=1) followed by two further articles in 2002, two in 2006 and one in 2008.

In decreasing order of scientific relevance, nine Randomized Clinical Trials were found (one in 2000, two in 2001, one in 2003, two in 2004, two in 2005 and one in 2008), plus one Cohort Study (2008), five Case-Control Studies (two in 2001, one in 2002, one in 2006 and one in 2007), five Case Series Studies (three in 2002, one in 2003 and one in 2007), three Case Studies (one in 2001 and two in 2003), while no studies at the Research in Animals level was found. Only one study for Specialist Opinion level was found (2002).

The results showed an increase in scientific output from the beginning of the twenty-first century on the theme of cognitive rehabilitation in the elderly with Alzheimer type dementia, while the highest levels of evidence were observed in the more recent publications.

Notably, among the studies analyzed, the association between pharmacologic therapy and cognitive rehabilitation was described at various different levels of scientific evidence since 2001.

The majority of researchers suggested that cognitive rehabilitation can better benefit patient rehabilitation when combined with other interventions such as pharmacologic treatment, interventions involving family members and environmental interventions. The latest studies demonstrating greater scientific evidence concluded that results of studies remain limited and that further investigation on the topic is needed.

## Discussion

The increased prevalence of dementias has driven the need for further research in this area. The results demonstrated an increase in scientific output from 2001 on the theme of cognitive rehabilitation in the elderly with Alzheimer type dementia.

The highest levels of evidence were observed in the more recent publications, thereby demonstrating the growing importance and concern in recent years of the scientific output addressing the care and assistance of patients with Alzheimer’s disease as well as of caregivers, family and health professionals involved with the disease. Furthermore, these findings support the development and growth of implementation of evidence-based practices across all fields of health research, so as to ensure more qualified and scientifically-based care and intervention.

Future studies are needed investigating cognitive rehabilitation in Alzheimer Disease patients, to pool and disseminate the aspects and concepts present among the highest levels of evidence, both in the context of data and information acquired from demented individuals, as well as towards implementing the best treatment interventions through well-informed clinical decisions.

More in-depth studies are warranted to build on the contributions made by these articles to cognitive rehabilitation and its applicability in caring for demented elderly.
